# Strategic and clinical insights from the rapid scale-up of over 200,000 specialty teleconsultations in remote and underserved Brazilian regions

**DOI:** 10.3389/fpubh.2026.1706130

**Published:** 2026-06-08

**Authors:** Tarso Augusto Duenhas Accorsi, Fernanda de Paula Nunes, Bruna Dayanne Reges Amaral, Marianne Pojali de Arruda, Bruna de Melo Souza, Ana Eliza Acerbi Sarti, Caroline Mendes Alves Borges, Cláudia Cristine Curcio, Karen Francine Köhler, Ari Stiel Radu Halpern, Flavio Tocci Moreira, Carlos Henrique Sartorato Pedrotti

**Affiliations:** Department of Telemedicine, Hospital Israelita Albert Einstein, São Paulo, Brazil

**Keywords:** eHealth policies, health policy, planning and management, health programs and plans, telemedicine, telemedicine for rural and remote areas

## Abstract

**Background:**

Large-scale provider-to-provider teleconsultation models remain underreported in low- and middle-income countries. Optimal strategies for scaling outpatient telemedicine and evaluating its impact on quality, efficiency, and patient experience are still being investigated.

**Methods:**

We conducted a retrospective descriptive implementation study of synchronous, video-based, provider-to-provider teleconsultations coordinated by the Telemedicine Department of Hospital Israelita Albert Einstein, São Paulo, Brazil. We summarized program reach, site implementation, consultation volume, specialty distribution, consultation disposition, no-show rate, schedule utilization, and post-consultation experience indicators.

**Results:**

A total of 365 service points were established, offering 12 specialties and performing 201,012 teleconsultations. Schedule occupancy remained at 99.8%. Among consultations, 79% resulted in follow-up within the same specialty, 16% were discharged from specialty care, and 5% were referred elsewhere. Common diagnoses included hypertension (3.9%), autism spectrum disorder (3.7%), joint pain (3.7%), ADHD (3.6%), and generalized anxiety disorder (3.3%). Net Promoter Scores were 91 for physicians and 85 for patients.

**Conclusion:**

A centralized, structured, multi-specialty telehealth strategy was feasible across remote locations, sustained high appointment occupancy, and effectively resolved or redirected 95% of cases. Both clinician and patient satisfaction were notably high, supporting the scalability of this model in underserved regions.

## 1 Introduction

The rapid expansion of telemedicine in ambulatory care, driven by the COVID-19 public health crisis, has deeply influenced the quality of care and patient safety. In particular, the widespread adoption of remote care has altered key factors such as communication, care team dynamics, and patient engagement, all essential for ensuring patient safety in an outpatient setting (1). The benefits of telehealth include triage, access to specialists, and the convenience of care delivery. However, many barriers have been identified that hinder its successful implementation and cost-effectiveness (2). Yet adoption is uneven; to achieve greater equity, policy reforms must target the barriers that disproportionately affect marginalized communities and the clinicians who serve them (3).

There are numerous challenges in implementing teleconsultations, with the most significant being the assurance of consultation quality and the practical feasibility of the recommended interventions (4, 5). In 2010, a landmark review of teleconsultation quality screened 1,593 titles and abstracts and ultimately included 80 heterogeneous systematic reviews. Of these, 21 reviews concluded that telemedicine is effective, 18 found the evidence promising but incomplete, while others deemed the evidence limited and inconsistent (6, 7).

Key themes include the challenges in conducting economic analyses of telemedicine, the benefits for patients, and telemedicine as a complex, ongoing collaborative process within unpredictable contexts. Telemedicine research is evolving, with new questions extending beyond clinical efficacy and cost-effectiveness. Larger controlled studies, more patient-centered outcomes and comprehensive economic analyses remain pressing needs (6). Telemedicine can offer numerous benefits in primary health care like expanding access to services in remote areas, reducing unnecessary referrals to secondary and tertiary levels (thus saving time and costs), and accelerating diagnosis and treatment with specialist support. A review found that teleconsultations in primary health care facilitated continuous access to care in underserved areas, reduced patient travel, and avoided inappropriate referrals without compromising the quality of care. In many contexts, teleconsultations have proven to be non-inferior to face-to-face visits in terms of clinical outcomes, with a potentially positive impact on efficiency and continuity of care (8, 9).

In February 2020, the Brazilian government launched a teleconsultation program for clinical specialties to serve populations in remote and underserved areas, employing a model where remote specialists consult collaboratively with local general practitioners attending to patients at primary care facilities. This initiative was implemented through the Institutional Development Support Program of the Brazilian Unified Health System (PROADI-SUS), a strategic partnership between the Ministry of Health and leading hospitals of excellence aimed at strengthening the public health system by expanding access, standardizing care delivery, and fostering innovation in specialized teleconsultation services. We hypothesized that centralizing telemedicine services and standardizing medical care implementation would lead to more access points while maintaining occupancy rates, ensuring efficient case resolution, and preserving high levels of user satisfaction.

This study aimed to outline the strategy for implementing these teleconsultations. Specifically, we sought to quantify the program's reach (the number of teleconsultation service points established and the total consultations performed) and to evaluate key performance indicators such as appointment occupancy rate, consultation outcomes (proportions of follow-up, referral, or discharge), and clinician/patient satisfaction as measures of the program's impact.

## 2 Methods

### 2.1 Study design and participants

We conducted a single-center retrospective study at the TM Center of Hospital Israelita Albert Einstein in São Paulo, Brazil, between February 2020 and May 2024, covering both the implementation and analytic teleconsultation windows. All patients referred by local primary care physicians for teleconsultation during the study period were eligible for inclusion, and no exclusion criteria were applied, as the analysis encompassed the entire population served by the program. Referrals for teleconsultation were made by primary care physicians based on their clinical judgment of patient need for specialist input. Essentially, any patient in the primary care clinic's waiting list who could benefit from specialist consultation was eligible for telemedicine referral, provided the case was not an emergency requiring immediate in-person intervention.

The study protocol along with the waiver of consent (based on the analysis of anonymized retrospective routine care data), was approved by the Review Board of Hospital Israelita Albert Einstein (registration number CAAE 77023923.2.0000.0071).

### 2.2 TeleAMES program

The TeleAMES (an acronym for the Portuguese translation of “Specialty Outpatient Clinics by Telemedicine”) program was conducted through PROADI-SUS (Program to Support Institutional Development of the Unified Health System), a collaborative initiative involving six leading private hospitals in Brazil and the Ministry of Health. Established in 2009, the program aims to support and enhance the public health system through projects focused on human resource training, research, technology assessment and incorporation, management, and specialized assistance as mandated by the Ministry of Health. TeleAMES provided specialized medical assistance in the North and Central-West regions of Brazil via telemedicine, and it utilized a single hospital for this purpose: Hospital Israelita Albert Einstein in São Paulo, Brazil.

### 2.3 Scope of action

The program was implemented across 10 Brazilian states, covering 5,460,076 km^2^ and spanning diverse biomes, including the Amazon Rainforest, Pantanal, and Cerrado. This extensive territory is marked by vast distances, limited infrastructure, and a high concentration of municipalities with low Human Development Index (HDI) scores, highlighting substantial barriers to healthcare access.

### 2.4 Planning

Municipalities were informed about the project directly through the Ministry of Health, which served as the central coordinating body of the PROADI-SUS initiative. The Ministry issued official invitations to eligible municipalities in the North and Central-West regions based on regional healthcare needs, waiting-list data, and strategic priorities previously mapped by federal authorities. These invitations were sent to state health secretariats and municipal health departments, accompanied by explanatory materials outlining the objectives, operational model, expected benefits, and the technical requirements for participation.

Enrollment followed a structured and standardized process. Municipalities that wished to participate submitted a formal request to the Telemedicine Center of Hospital Israelita Albert Einstein. This request included: identification of the primary care facility selected to host the teleconsultation room, including address and National Registry of Health Establishments (CNES) number; specialty-specific waiting-list reports demonstrating local demand; and information on service capacity, such as the number of registered families and population coverage. After receiving the request, the Telemedicine Center verified the documentation, assessed infrastructure readiness, and, when necessary, provided guidance to ensure compliance with equipment and connectivity requirements. Once approved, the municipality proceeded to staff training, installation of equipment, and activation of the teleconsultation schedule.

As an initial plan, the program aimed to implement 232 teleconsultation points (120 in the North region and 112 in the Central-West). However, as the project gained momentum, additional municipalities that were not part of the original plan requested to join. This expansion led to a total of 385 teleconsultation sites established by 2024, of which 365 remained active at the end of the study period.

In municipalities that applied after the program's initial rollout, the required data (current waiting list, waiting list from 15 days and 1 month prior, and number of registered patients at the target primary care facility) were generally submitted quickly and effectively. Most municipalities responded within a few days after receiving the request, as participation in TeleAMES was strongly motivated by the need to reduce long specialty-care waiting lists. The standardized data template provided by the Telemedicine Center also facilitated rapid submission. While occasional delays occurred—mainly in municipalities with limited administrative capacity or non-digital waiting-list systems—these were exceptions rather than the norm. Overall, the process was efficient and rarely slowed implementation timelines.

The selected local room was required to meet the standards for medical consultations, ensuring privacy, adequate lighting, and an environment free from external noise.

It was also essential to recognize the need for adequate infrastructure for implementation, including the recommended equipment kit: a laptop, webcam, and a microphone with speaker. All participating units must meet the following hardware and network requirements:

- Laptop or Computer: Laptop/Computer: 8th generation Intel Core i5 processor (or higher); 8 GB RAM (or higher); speakerphone (Jabra 410 recommended); HD webcam (Logitech C920 recommended); operating system Windows 7, Windows 10, or macOS 10.14 (or higher); and supported browsers (Google Chrome v94+ or Mozilla Firefox v91+ with WebRTC support).
- Network: TCP ports 443, 5061, 8100 open; UDP ports 10,000–60,000 (recommended) or at least 35,000–35,999 open outbound, with replies allowed from specified domains/IPs (including ^*^.intouchhealth.com, ^*^.intouchcustomer.com, ^*^.intouchreports.com, ^*^.intouchconnect.net, ^*^.visitnow.org, ^*^.telemedicinaeinstein.com.br; IPv4 range 170.176.128.0/18; IPv6 range 2620:12C:9000::/40).

These equipment and connectivity requirements were mandated to ensure high-quality, uninterrupted teleconsultations. Adherence to these standards minimized technical difficulties (e.g., poor audio-video quality or disconnections) that could undermine the effectiveness of the consultation.

The two-hour training session offered to local teams was delivered online by the Telemedicine Center of Hospital Israelita Albert Einstein and followed a standardized curriculum to ensure uniform implementation across all sites. The training targeted four key professional groups: the scheduling clerk (usually a nurse), the local primary care physicians, the IT technician, and the municipal regulatory-center coordinator. The curriculum covered both operational and clinical components.

First, participants received an overview of the TeleAMES program, including objectives, workflow structure, and roles and responsibilities of each team member. The technical segment included step-by-step instruction on accessing the telemedicine platform, initiating and conducting video consultations, uploading and retrieving documents, and completing electronic forms. IT personnel received additional guidance on network requirements, audio-video configuration, troubleshooting connectivity issues, and performing the mandatory 30-min pre-launch connectivity test.

The clinical segment focused on referral criteria, appropriate case selection, and how to prepare patients for the virtual encounter. Training also emphasized professional conduct, documentation standards, privacy requirements, and how to operationalize the specialist's recommendations after the teleconsultation (prescriptions, test orders, follow-up arrangements).

At the end of the session, teams were provided with access to the Virtual Library—an online repository containing instructional videos, protocol documents, troubleshooting guides, and specialty-specific referral prerequisites—allowing asynchronous review and continued support after implementation.

Following the initial training, an electronic repository is provided through the Einstein Telemedicine System, currently referred to as the Virtual Library. This repository houses all training materials, including instructional videos and PDFs detailing system usage. Additionally, it contains clinical protocols developed by the heads of each specialty, critical communications, FAQs, and the prerequisites for referring patients to specialized care.

### 2.5 Principles of teleconsultation

Appointments were scheduled Monday through Friday, 8:00 a.m. – 7:00 p.m. Brasília time, each lasting 30 min. To optimize patient care, coordination with the Regulatory Center was essential to ensure patients on the waiting list were seen and any backlog in demand was prioritized. The on-site team scheduled appointments according to the times provided by the Hospital Israelita Albert Einstein Telemedicine Center. Teleconsultations were synchronous, video-based encounters linking a remote specialist with a general practitioner and patient co-located at a primary-care facility; each 30-min slot followed a standard workflow coordinated by the municipal regulatory center.

The specialist provided written treatment recommendations on an official form sent to the on-site team. The on-site physician then issued prescriptions, ordered necessary tests, provided patient education, and managed follow-up. If needed, follow-up appointments with the specialist could be arranged. However, ongoing follow-up via telemedicine was generally not recommended, and patients were advised to return to the primary-care facility or be referred for tertiary evaluation if necessary.

The virtual assessment records were maintained by the Telemedicine Center, fully electronic and accessible to authorized staff. The patient's records at the Primary Health Facility were managed using the local system (which was sometimes not yet digital) and were updated by the on-site general practitioner, incorporating the specialist's report.

Locations approved by Local Health Administration and Telemedicine Center for project implementation prepared the service area with the necessary local equipment. The municipality designated the primary-care facility for the Telemedicine project, ensuring it was integrated with Primary Health Care.

After local approval, Einstein staff installed the equipment, performed a 30-min connectivity test, and created user accounts. A project team was defined at each site—comprising a scheduling clerk (preferably a nurse), the local physician(s), an IT technician, and the municipal regulatory-center lead—who then completed a two-hour online training session and booked the first specialty visits.

Coordination with the municipal regulatory center was essential to address local demand, manage referrals from Primary Care, and ensure the patient returned to the family physician for follow-up after the specialist consultation.

The methodology outlines that information is received from municipalities interested in implementing the project. “Official” sites were pre-selected by health authorities (North: Ministry of Health by ascending HDI; Central-West: State Health Departments and Federal District). Waiting-list data were therefore unavailable for many official locations.

Municipalities applying later provided: (i) current waiting list for the covered specialties, (ii) waiting list 15 days and one month prior, and (iii) number of registered patients at the target primary-care facility. Service-capacity data were not requested.

Patient referral to teleconsultation followed a structured yet pragmatic workflow embedded within routine primary care practice. Referrals were initiated by local primary care physicians based on clinical judgment, primarily targeting patients already registered on specialty care waiting lists who were deemed suitable for remote specialist evaluation and did not require urgent in-person assessment. The process was coordinated in conjunction with municipal regulatory centers, which were responsible for prioritizing cases according to local demand and organizing scheduling within the available teleconsultation capacity. This approach reflects a real-world implementation strategy, designed to optimize access to specialist care in resource-constrained settings while maintaining operational feasibility across geographically diverse regions.

Schedule occupancy was defined as the number of completed teleconsultations divided by the number of consultation slots released for booking in the corresponding period. Because slot release was operationally adjusted according to local demand and specialist availability, this metric should be interpreted as a utilization indicator rather than as a direct measure of unmet need or program effectiveness.

Repeat specialist teleconsultation was scheduled when clinically indicated, not as default long-term policy.

### 2.6 Specialties provided

TeleAMES initially provided seven specialties — adult cardiology, adult endocrinology, adult pulmonology, adult rheumatology, adult neurology, pediatric neurology, and psychiatry — and subsequently expanded to a total of 12 with the inclusion, after January 2024, of pediatric endocrinology, adult infectious diseases, adult gastroenterology, pediatric gastroenterology, and pediatrics.

### 2.7 Quality meetings

Monthly meetings aligned project objectives, shared operational metrics, and provided feedback. Each specialty team also held weekly record audits with its medical coordinator. Eight virtual classes and periodic case conferences were delivered across specialties. Each state received monthly reports detailing consultation counts, cancellations, logbook summaries, and system updates. This regular feedback loop ensured timely data submission from all sites and allowed the central team to continuously monitor performance and address issues quickly.

As part of routine quality monitoring, physicians and patients were invited to complete a post-consultation recommendation survey using a 0–10 scale. Net Promoter Score (NPS) was calculated separately for physicians and patients as the percentage of promoters (scores 9–10) minus the percentage of detractors (scores 0–6), with passive respondents (scores 7–8) retained in the denominator. Survey adherence was defined as the number of completed surveys divided by the number of eligible consultations. Because survey completion was voluntary, these findings were interpreted as experience indicators and may be affected by non-response and social-desirability bias.

### 2.8 Statistical analysis

All analyses were descriptive and were performed using IBM SPSS Statistics for Windows, version 22.0, and R, version 4.3.0. Because the study included the full set of teleconsultations recorded during the analytic period, no formal sample-size calculation was performed. Categorical variables were summarized as absolute counts and percentages. Continuous variables were summarized as means and standard deviations or medians and interquartile ranges, as appropriate to their distribution. For key operational proportions, including occupancy, no-show rate, consultation disposition, and survey adherence, 95% confidence intervals were calculated. The definitions of all outcomes and denominators are provided in Table 1.

**Table 1:** Operational outcomes, analytic definitions, and denominators used in the TeleAMES evaluation.

Domain	Outcome/ indicator	Operational definition	Denominator used for analysis
Implementation reach	Implemented teleconsultation site	Primary health care facility in which the TeleAMES infrastructure was installed, the local team was trained, user access was configured, connectivity was validated, and at least one specialty teleconsultation schedule was made available.	All municipal primary health care facilities approved and activated for the TeleAMES program during the implementation period.
Implementation reach	Active teleconsultation site	Implemented site that remained operational and available for scheduling at the end of the analytic period.	All implemented TeleAMES sites.
Implementation reach	Completed teleconsultation	Synchronous, video-based provider-to-provider specialty encounter in which the remote specialist evaluated the case with the patient and local physician co-located at the primary care facility, generated an end-of-encounter recommendation, and the encounter was recorded in the institutional telemedicine system.	All teleconsultation appointments scheduled or released for booking during the analytic period, as specified for each utilization analysis.
Implementation reach	Patients served	Unique patients who completed at least one TeleAMES specialty teleconsultation during the analytic period.	All patients referred by local primary care physicians and recorded in the TeleAMES database.
Implementation reach	Specialties provided	Clinical specialties for which structured synchronous teleconsultation slots were offered by the TeleAMES program.	All specialties formally incorporated into the program during the study period.
Utilization	Schedule occupancy rate	Proportion of released appointment capacity that resulted in a completed teleconsultation. The metric was used as an operational utilization indicator because slot release was adjusted according to local demand and specialist availability.	Number of consultation slots released for booking in the corresponding period. Numerator: completed teleconsultations.
Utilization	No-show rate	Proportion of scheduled teleconsultations not completed because the patient did not attend the appointment at the primary care facility.	All scheduled teleconsultations for which patient attendance status was available. Numerator: scheduled teleconsultations missed because of patient non-attendance.
Utilization	Site withdrawal after implementation	Implemented site that discontinued participation after activation of the TeleAMES workflow.	All implemented TeleAMES sites. Numerator: sites that withdrew after implementation.
Utilization	Pre-implementation withdrawal	Municipality or site that withdrew after initial approval or planning but before full activation of the teleconsultation workflow.	All municipalities or sites that entered the pre-implementation process. Numerator: sites that withdrew before activation.
Consultation disposition	Same-specialty follow-up	End-of-encounter specialist recommendation indicating that the patient should be scheduled for a subsequent teleconsultation in the same specialty because continued specialist input was clinically indicated.	All completed teleconsultations with a recorded specialist disposition. Numerator: encounters assigned to same-specialty follow-up.
Consultation disposition	Discharge from specialty care	End-of-encounter specialist recommendation indicating that the case could be managed in primary care without further planned follow-up in that specialty, based on the specialist assessment and written recommendations.	All completed teleconsultations with a recorded specialist disposition. Numerator: encounters discharged from specialty care.
Consultation disposition	Referral to another specialty or level of care	End-of-encounter specialist recommendation indicating that the patient required evaluation by another specialty or escalation to another point of care, including in-person tertiary assessment when clinically necessary.	All completed teleconsultations with a recorded specialist disposition. Numerator: encounters referred to another specialty or level of care.
Case-mix	Specialty distribution	Proportion of completed teleconsultations attributable to each specialty service.	All completed teleconsultations. Numerator: completed teleconsultations in each specialty.
Case-mix	Most frequent ICD-10-coded diagnoses	Distribution of the most commonly recorded diagnostic categories coded using the International Classification of Diseases, 10th Revision, in completed teleconsultations.	All completed teleconsultations with at least one recorded ICD-10 code. Numerator: encounters assigned to each ICD-10 diagnostic category.
User experience	Physician Net Promoter Score	Post-consultation recommendation indicator calculated among responding physicians as the percentage of promoters (score 9-10) minus the percentage of detractors (score 0-6) on a 0-10 recommendation scale; passive respondents (score 7-8) were retained in the denominator.	All completed physician post-consultation surveys. Numerator components: promoters and detractors among responding physicians.
User experience	Patient Net Promoter Score	Post-consultation recommendation indicator calculated among responding patients as the percentage of promoters (score 9-10) minus the percentage of detractors (score 0-6) on a 0-10 recommendation scale; passive respondents (score 7-8) were retained in the denominator.	All completed patient post-consultation surveys. Numerator components: promoters and detractors among responding patients.
User experience	Survey adherence	Proportion of eligible completed teleconsultations for which the corresponding post-consultation experience survey was completed.	All completed teleconsultations eligible for the physician or patient survey, respectively. Numerator: completed surveys.

ICD-10, International Classification of Diseases, 10th revision; NPS, Net Promoter Score; TeleAMES, Specialty Outpatient Clinics by Telemedicine. All indicators were summarized descriptively. For operational proportions, 95% confidence intervals were calculated as specified in the statistical analysis plan.

Primary study outcomes were implementation reach (implemented sites, active sites, completed teleconsultations), utilization (occupancy and no-show rate), consultation disposition (same-specialty follow-up, discharge from specialty care, referral to another specialty), and user experience (physician and patient NPS). Occupancy was defined as completed teleconsultations divided by released consultation slots. No-show rate was defined as scheduled consultations not completed because the patient did not attend. Consultation disposition was assigned from the specialist's end-of-encounter recommendation.

## 3 Results

Initially, we planned to establish 120 implementation points in the North Region. However, as the strategy gained traction, municipalities initially excluded from the plan requested inclusion, leading to additional implementation points. A similar expansion occurred in the Central-West Region, where the initial target of 112 points also grew. These adjustments produced the current total of 365 implemented points.

**Figure 1:**
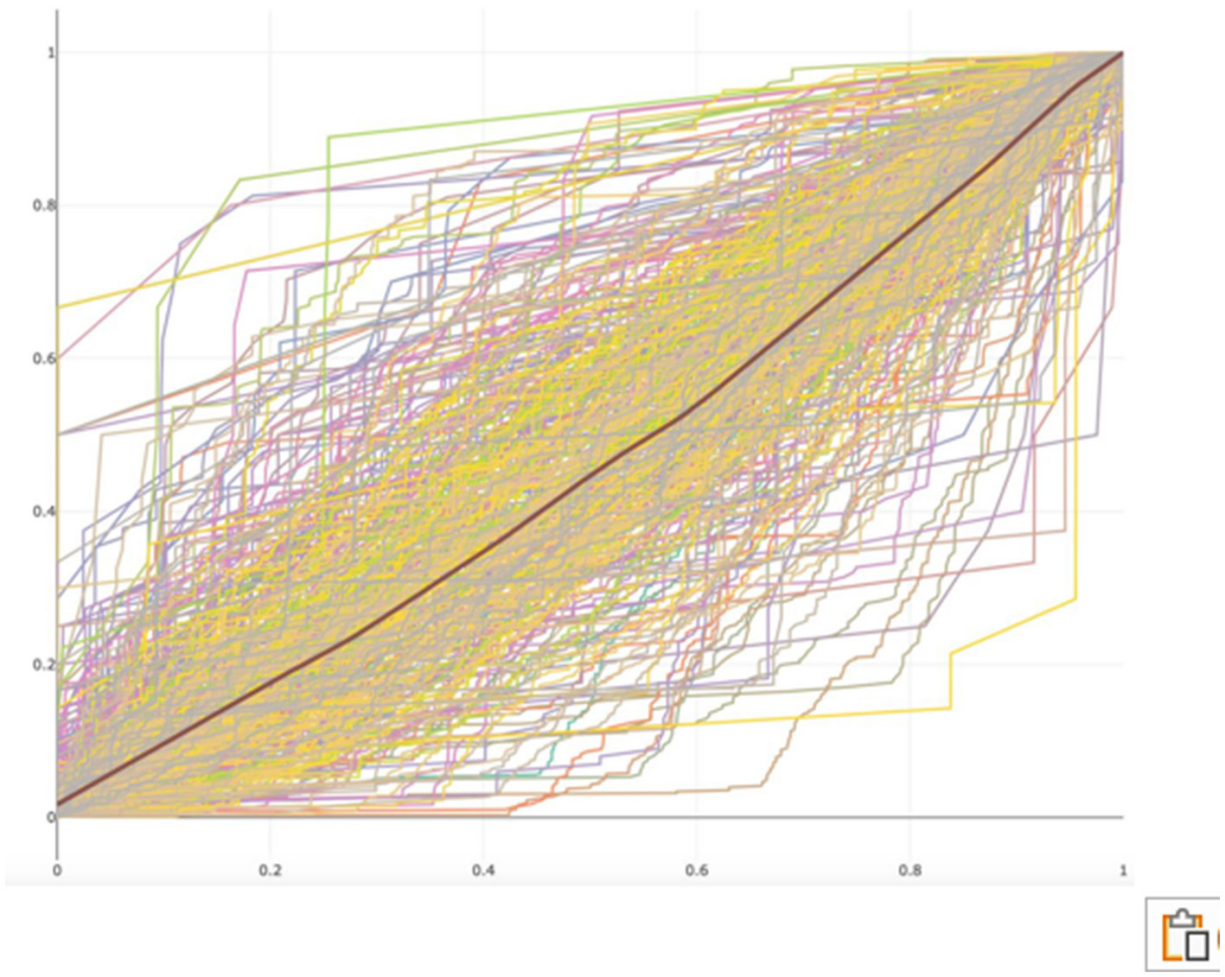
Normalized cumulative teleconsultation growth across service points.

Analysis of teleconsultation growth, normalized by implementation start date and initial volume for each site, revealed marked heterogeneity in adoption trajectories (Figure 1). While some sites showed rapid early uptake followed by plateauing, others exhibited gradual but steady expansion. Despite heterogeneity among sites, the aggregated curve was approximately linear over the study period. These findings suggest that while site-specific factors influenced the pace of adoption, the overall program maintained consistent expansion dynamics across the diverse regions served.

**Figure 2:**
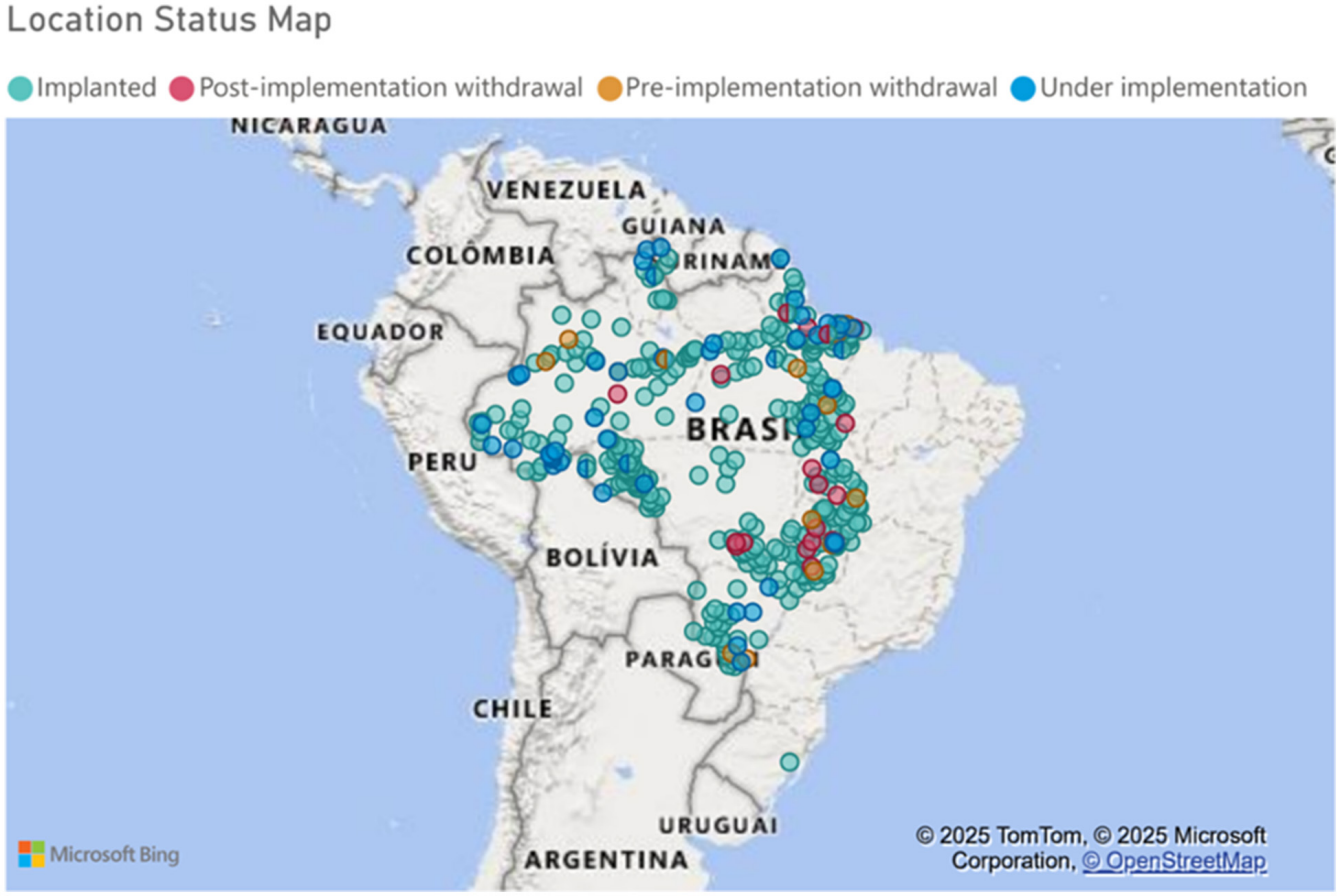
Distribution of teleconsultation points across primary health care facilities.

Between 2021 and 2024, 201,012 teleconsultations were completed, with monthly volumes ranging from 7,576 to 13,991 in the most recent months. A total of 147,937 patients were served across 385 implemented primary-care facilities, 365 of which remained active. Twenty sites withdrew after implementation and 14 withdrew beforehand (Figure 2). The no-show rate was 12.7%. Engagement strategies included SMS reminders, phone calls, and feedback dashboards. These initiatives were intended to improve attendance and engagement; for example, prior studies indicate that automated reminders can substantially reduce patient no-show rates.

**Figure 3:**
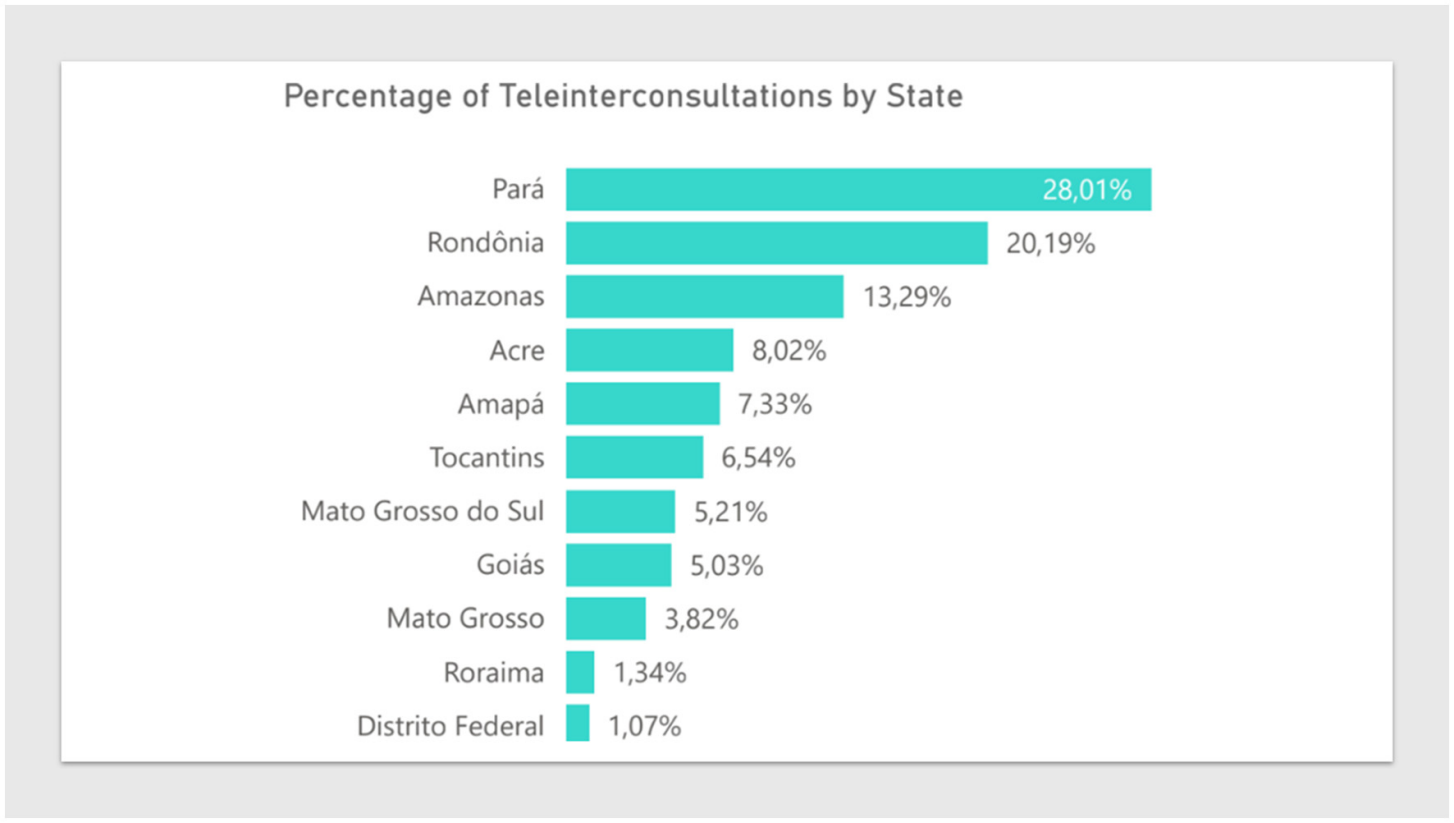
Distribution of the number of services provided by states in Brazil.

Most teleconsultations occurred in Pará (28.0%), Rondônia (20.2%), Amazonas (13.3%), Acre (8.0%), and Amapá (7.3%) (Figure 3).

Encounters ended in scheduled same-specialty follow-up (79–88%), referral to another specialty (9–16 %), or discharge from specialty care (9–12%), ranges referencing across different specialties.

**Figure 4:**
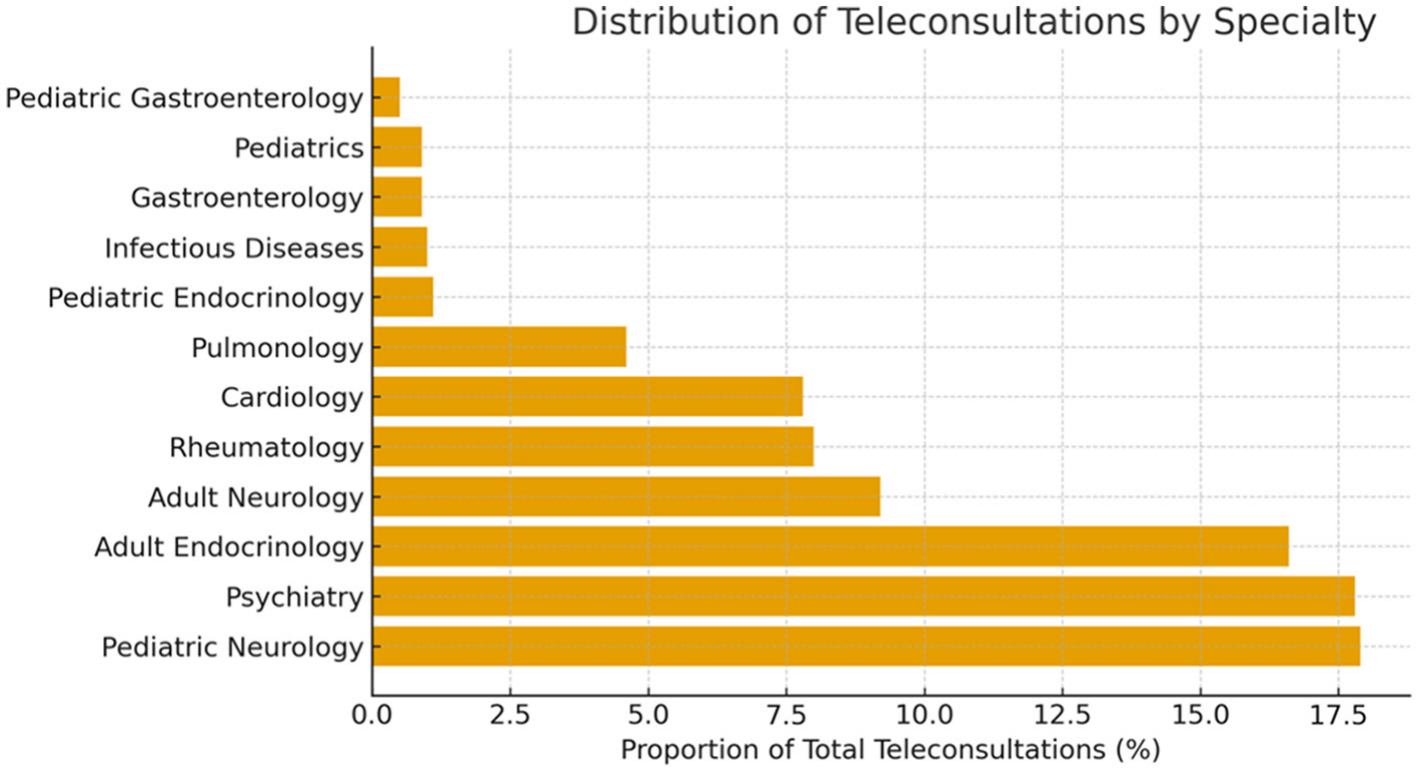
Distribution of teleconsultations by specialty.

Pediatric neurology (17.9 %), psychiatry (17.8 %), and adult endocrinology (16.6 %) accounted for over half of all visits. Lower-volume services included adult pulmonology (4.6%), pediatric endocrinology (1.1%), adult infectious diseases (1.0%), pediatrics (0.9%), and pediatric gastroenterology (0.5%) (Figure 4).

Overall, 2,336 physicians submitted teleconsultation requests between 2021 and 2024. Representation was highest in Pará (482), Amazonas (422), and Rondônia (418), and lowest in Roraima (65). Among consulting specialists, psychiatry (44), pediatric neurology (42), cardiology (35), adult neurology (34), and endocrinology (32) dominated.

**Figure 5:**
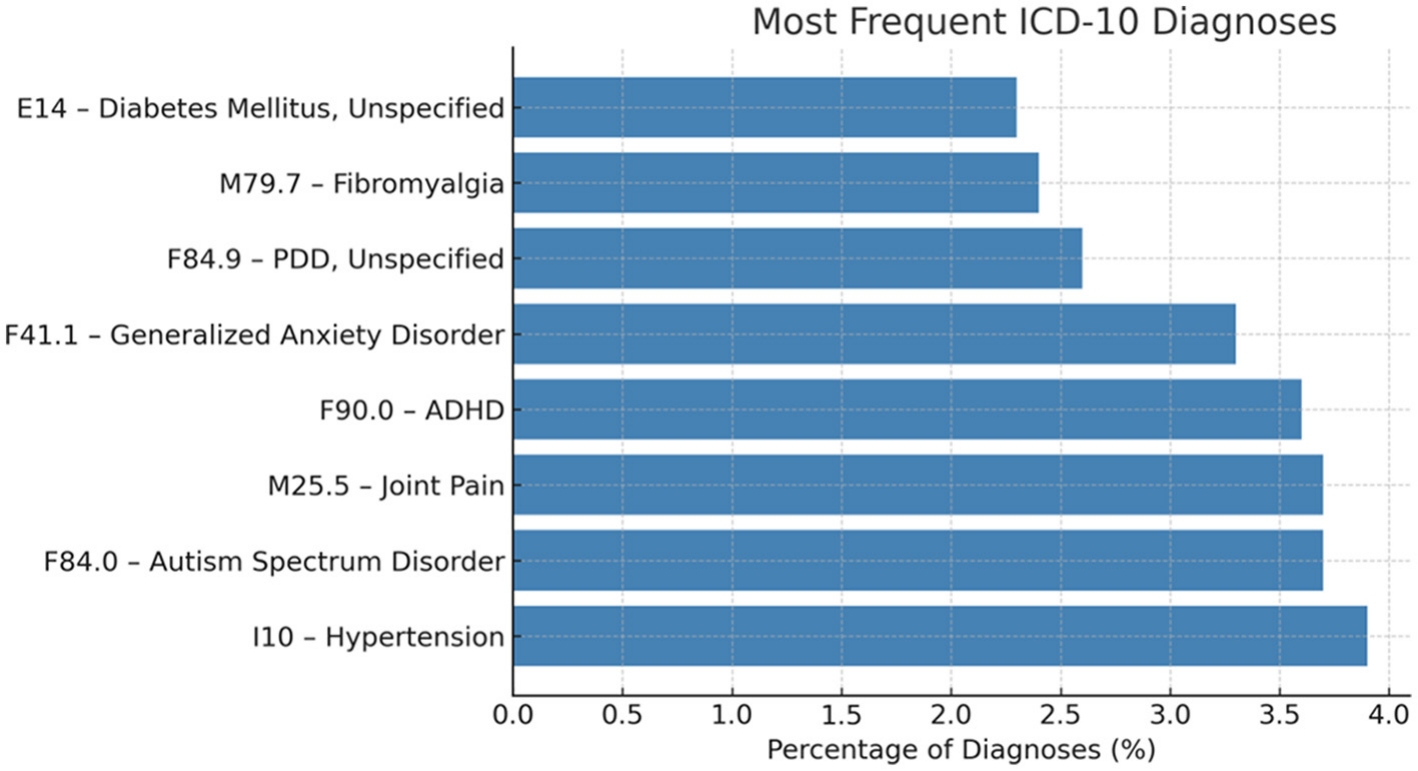
Main diagnoses coded using ICD-10.

The most prevalent ICD-coded diagnoses were essential hypertension (3.9%), childhood autism (3.7%), joint pain (3.7%), attention-deficit/hyperactivity disorder (3.6%), generalized anxiety disorder (3.3%), unspecified pervasive developmental disorder (2.6%), fibromyalgia (2.4%), and unspecified diabetes mellitus (2.3%) (Figure 5).

Mean schedule-occupancy rate was 99.8%; every specialty exceeded 97%, and neurology, pediatric neurology, pediatrics, psychiatry, and rheumatology reached 100%. The target occupancy rate was 100%, and the target for planned follow-up was 82%.

Net Promoter Score (0–100 scale) averaged 91 for physicians and 85 for patients, with monthly survey-completion (“adherence”) of 86% and a consolidated three-year adherence of 85%. Net Promoter Scores above 70 are considered excellent (10).

Work-hour allocation showed that nursing accounted for the largest share (1,340 h), followed by Project/Management (880 h), Support (220 h), Coordination (183 h), Financial (110 h), and Pharmacy (60 h).

These findings underscore the wide uptake and operational efficiency of TeleAMES: high engagement from patients and physicians, strong demand for subspecialty care, and an infrastructure capable of sustaining near-full capacity utilization.

## 4 Discussion

Implementing the TeleAMES program represents a significant step toward embedding telemedicine in Brazil's public healthcare system, particularly for remote and underserved populations. According to a March 2023 Ministry publication, Brazil spans 8,510,418 km^2^, making it one of the largest countries in the world. The North and Central-West regions account for about 45% and 18.5% of this territory, respectively, which presents challenges in healthcare delivery due to vast geography and an uneven physician distribution. Although the physician-to-population ratio increased from 1.15 per 1,000 inhabitants in the 1980s to 2.41 by 2022, regional disparities persist. The Southeast shows the highest density (3.39), whereas the North and Northeast have much lower ratios, such as Pará's 0.97. This imbalance partly reflects migration, as newly trained doctors relocate to urban centers for better opportunities. Over the past decade no capital lost doctors, yet some metropolitan and rural municipalities did, underscoring the difficulty of retaining professionals in underserved areas (11).

Our findings highlight the feasibility of teleconsultations in enhancing access to specialized care while ensuring high operational efficiency and user satisfaction. Rooted in the Brazilian Unified Healthcare System (SUS) principles of equity, universality, and comprehensiveness, programs like TeleAMES may shorten waiting times, reduce patient transfers, and transfer knowledge from centers of excellence. Furthermore, the project aligns with the goals of healthcare planning initiatives, which aim to transform the culture of healthcare organizations by emphasizing patient safety, implementing improvements, strengthening Primary Health Care, and contributing to the organization of the Health Care Network within the SUS.

This retrospective observational study was designed to describe implementation and service-utilization patterns. Because the program was rolled out operationally and no concurrent control group or standardized preimplementation baseline was available for all municipalities, the findings should be interpreted as descriptive evidence of program reach and operation rather than as evidence of comparative effectiveness or causal impact.

The near-perfect agenda occupancy (99.8%) and 238,708 completed visits illustrate wide acceptance, echoing earlier work showing telehealth's ability to overcome distance barriers (6). This near-perfect utilization met the program's goal of fully occupying available consultation slots, indicating that capacity was optimally matched to demand. Nevertheless, uneven uptake across states signals that local infrastructure, governance, and digital readiness modulate adoption (12).

A key aspect of the program's success was its structured implementation approach, which included careful planning, established infrastructure requirements, and ongoing professional training. The required use of specific hardware and network configurations ensured standardized, high-quality teleconsultation experiences, reducing the technical limitations often faced in telemedicine deployments (13). Moreover, embedding specialists into primary-care encounters furthered collaborative decision-making and continuity (14). Nonetheless, even with these enabling factors, we encountered challenges during implementation. Some remote sites initially faced unstable internet connectivity, which could disrupt sessions and erode provider confidence. We mitigated this by rigorously applying the technical requirements and conducting connectivity tests at each site. In parallel, a number of local clinicians were hesitant about the teleconsultation model — a barrier observed in other telehealth initiatives. To overcome this skepticism, we provided comprehensive training and highlighted early positive outcomes, which helped build trust and buy-in among the healthcare teams. The specialty distribution data indicate that pediatric neurology, psychiatry, endocrinology, and adult neurology represented the highest volumes of teleconsultations. This trend highlights the growing burden of neurodevelopmental disorders, psychiatric conditions, and chronic illnesses that necessitate ongoing specialist care follow-up (15). Telepsychiatry's strong uptake aligns with evidence that it improves access and outcomes where psychiatrists are scarce (16, 17).

Despite these successes, some challenges persist. A significant finding requiring further investigation is the high proportion of consultations resulting in planned same-specialty follow-up (79–88%). While indicating successful initial access, this suggests many conditions managed required ongoing specialist oversight, potentially straining program capacity and raising questions about the long-term role of teleconsultation vs. transitioning care back to strengthened primary care following specialist guidance. Future analyses could explore factors associated with requiring prolonged follow-up vs. discharge, particularly in cases that require frequent treatment adjustments or in-person physical examination (18). Also, the modest discharge rate (9–12%) emphasizes the need to integrate teleconsultation findings into the wider healthcare system, ensuring that patients receive appropriate in-person evaluations when necessary. Another limitation is the disparity in telemedicine adoption across various states. While Pará, Rondônia, and Amazonas accounted for the highest number of teleconsultations, other states, such as Roraima, showed lower participation rates. These discrepancies may be due to differences in local infrastructure, internet connectivity, and regional healthcare access policies (19). Moreover, our study did not include a concurrent control group or pre-telemedicine baseline for comparison. This limitation makes it challenging to directly quantify TeleAMES's impact relative to standard care. Future evaluations should consider incorporating baseline data or control cohorts to more rigorously assess the specific benefits of teleconsultation programs. Although the TeleAMES care model incorporated safety-oriented operational features, such as specialist documentation, local physician co-presence, and defined escalation pathways, patient-safety outcomes were not systematically captured in the analytic database. The present study therefore cannot quantify diagnostic safety, medication-related incidents, or post-consultation adverse events.

The referral-based nature of patient inclusion introduces a potential for selection bias. As referrals were made at the discretion of local primary care physicians and influenced by regional healthcare organization, infrastructure availability, and waiting-list dynamics, the study population may not be fully representative of the broader patient population requiring specialist care. Additionally, heterogeneity across participating sites may have contributed to variability in case-mix and clinical complexity. Therefore, the findings should be interpreted within the context of a pragmatic, real-world telemedicine implementation rather than a standardized or randomized selection framework.

The high occupancy observed in the program indicates sustained utilization of released consultation capacity; however, because schedule availability was operationally managed, this metric should not be interpreted in isolation as evidence of comparative effectiveness or of the full magnitude of underlying specialty-care demand.

In some specialties TeleAMES accounted for up to 10% of all consultations statewide, consistent with global trends in primary-care telemedicine. A US study of 2.36 million visits found telemedicine used in 51% of contacts, with slightly higher short-term follow-up after video/telephone visits but no surge in emergency-department use (20). Another cohort study examined the differences between telemedicine and in-person primary care visits, particularly in physician prescribing, orders for lab tests or imaging, and follow-up care. It included 1,131,722 patients and 2,178,440 appointments, with 14% conducted via telemedicine. Results showed that telemedicine visits, both video and telephone, had lower rates of medication prescriptions and orders for laboratory tests or imaging compared to in-person visits. Follow-up visits within seven days were slightly higher for telemedicine, but there were no significant differences in emergency department visits or hospitalizations. The study concluded that telemedicine offers a convenient and effective alternative for accessing primary care without increasing adverse health outcomes (21).

By contrast, a comprehensive cross-sectional study revealed that patients exhibit a preference for in-person consultations over telemedicine appointments (22). These mixed findings highlight the importance of tight integration between virtual and conventional services.

Other challenges need to be addressed to enhance the efficiency and acceptance of telemedicine comprehensively. A study involving 75,947 patients and 1,155 clinicians, across 137,846 scheduled video encounters, found that 10% of these encounters experienced audio-video communication failures. Factors associated with a shift to telephone consultations included lower clinician comfort with technology [odds ratio (OR), 0.15; 95% CI, 0.08–0.28], older patient age (66–80 years: OR, 0.28; 95% CI, 0.26–0.30), lower patient socioeconomic status, including limited access to high-speed internet (OR, 0.85; 95% CI, 0.77–0.92), and minority racial or ethnic background (Black or African American: OR, 0.75; 95% CI, 0.69–0.81). The study highlights the importance of addressing patient support and equity as policymakers consider expanding telehealth coverage (23). In addition to patients with limited proficiency in the local language, older individuals, those from lower socioeconomic backgrounds, and minority groups also tend to use telemedicine less frequently (24).

Beyond digital access, telehealth services must adhere to established quality standards, as inconsistent care quality can disproportionately impact vulnerable populations, exacerbating existing healthcare disparities. As telemedicine continues to reshape the clinical landscape, it is crucial to promote health equity by advocating for digital equity. We must proactively identify and address potential disparities to prevent them from widening as telemedicine becomes more prevalent (25).

In USA, State regulations require that a physician offering E-health services be licensed in the state where the patient receives care (26). In Brazil, Federal Law No. 14.510/2022 authorizes telemedicine nationwide, allowing healthcare services across state borders while requiring compliance with ethical standards and data protection laws (27).

Medical payment fraud is a risk in telemedicine, where institutions may abuse personal data to submit false insurance claims. The increasing use of telemedicine heightens this vulnerability, alongside risks from data breaches by hackers. Additionally, medical errors may occur due to the limitations of telemedicine, particularly from misdiagnosis, though the risk is mainly confined to non-fatal conditions. There are also concerns about unlicensed medical practices, as telemedicine could facilitate access to unregulated practitioners, both locally and internationally, including the dark web. The advancement of telemedicine introduces new challenges regarding medical devices, which must meet stringent reliability standards. Issues such as illegal manufacturing, use of unauthorized devices, and software vulnerabilities pose threats. Telemedicine also raises the risk of data abuse, as it collects and stores highly sensitive medical information that may be targeted by hackers or mishandled by medical professionals. In the US, healthcare providers have faced ransomware attacks, highlighting the ongoing threat to data security. Lastly, drug abuse is another concern, as telemedicine makes it easier to purchase medications, which could lead to misuse, particularly for sensitive drugs. Countries like China are tightening regulations on online drug sales to address this issue, though balancing accessibility with control remains a challenge (28).

It is crucial to emphasize that professional contracts, quality standards, and institutional protocols applied in face-to-face consultations must be adapted and strictly followed in virtual consultations. This was made possible through the centralization of the telemedicine actions referenced in this project.

To continue the project and expand medical care access for the population, Telemedicine Center of Hospital Israelita Albert Einstein will provide 490,000 slots for scheduling teleconsultations. It is important to note that, in accordance with Resolution No. 2,314 of the Federal Council of Medicine, dated April 20, 2022, Article 7, “Teleconsultation is the exchange of information and opinions between physicians, facilitated by Digital Information and Communication Technologies (TDICs) (3), with or without the presence of the patient, for diagnostic, therapeutic, clinical, or surgical assistance.” The project will also promote the establishment of additional telemedicine points. For this expansion, the municipality or administrative region must ensure that a primary health facility in the locality meets the necessary prerequisites and minimum infrastructure requirements for the project's proper functioning and that there is a significant unmet demand, such as a long waiting list at the locality's regulatory center. After this assessment, a request should be submitted to the Municipal Health Department to formalize the application to the State Health Department, which will then forward the request by letter to Telemedicine Center. The project aims to expand to up to 600 implemented points. Telemedicine Center and the project team will conduct a thorough analysis based on criteria such as the suitability of the requested location for Primary Health Care Unit under the Family Health Strategy, distance from the capital, utilization of the project at other implemented locations, waiting list length, and other relevant factors. This analysis will be aligned with the Ministry of Health and the National Council of Health Secretaries to ensure follow-up at the requested location. Once these entities agree, the process of implementing the project in the municipality can begin. Additionally, the project will focus on reducing the no-show rate, with a target of achieving a 15% no-show rate, and maintaining high service quality by keeping both physician and patient satisfaction survey results within or above the designated quality zone. Evidence from the literature shows that SMS reminders, direct telephone outreach, and periodic performance dashboards shared with municipal coordinators can significantly improve attendance and reduce no-show rates. For example, Hasvold and Wootton (29) demonstrated that SMS and telephone reminders increased appointment adherence across multiple healthcare settings, while Adams et al. (10) emphasized how structured feedback loops and transparent performance metrics enhance provider engagement and program sustainability. These engagement strategies were therefore incorporated into TeleAMES not only as operational tools but as evidence-based mechanisms to support participation, improve attendance, and maintain high utilization rates.

Future telemedicine expansions should focus on addressing these regional inequalities by strengthening digital health infrastructure and providing targeted training programs for healthcare professionals in lower-adopting regions. Furthermore, although only 34 sites withdrew, this signals sustainability issues—workforce turnover, funding gaps, or technical obstacles (30). Ongoing federal and institutional support is vital. The high three-year Net Promoter Score of 85 indicates strong engagement, but qualitative work must unpack drivers of satisfaction and reasons for attrition (31).

In summary, TeleAMES demonstrates that a centralized, structured, multi-specialty telehealth model was feasible at scale across remote locations and showed sustained operational utilization and favorable experience indicators. Because the study was retrospective and lacked a concurrent comparator or standardized baseline across sites, the findings should be interpreted as descriptive program evidence rather than as comparative effectiveness or safety evidence. Future efforts should tackle regional inequities, reinforce long-term sustainability, and clarify telemedicine's optimal role in chronic-care pathways.

## Data Availability

The raw data supporting the conclusions of this article will be made available by the authors, without undue reservation.
